# The effect of malotilate, a derivative of malotilate and a flavenoid on eicosanoid production in inflammatory bowel disease in rats

**DOI:** 10.1155/S0962935193000092

**Published:** 1993

**Authors:** A. P. M. van Dijk, J. H. P. Wilson, F. J. Zijlstra

**Affiliations:** 1 Department of Pharmacology Erasmus University Rotterdam P.O.Box 1738 Rotterdam 3000 DR The Netherlands; 2 Department of Internal Medicine II Erasmus University Rotterdam P.O.Box 1738 Rotterdam 3000 DR The Netherlands

## Abstract

Acetic acid induced colitis in rats was used to investigate the effects of malotilate, a drug which has been shown to inhibit 5-1ipoxygenase in human macrophages, the malotilate derivate ZY16268 and the flavenoid ZY16369 on the eicosanoid production and the colonic morphology in inflammatory bowel disease. Acetic acid produced an acute inflammatory response in the colon, associated with a markedly raised inflammation score (15.8 vs. < 0.5), based on a seven-scaled scoring system which includes observation of haemorrhage, submucosal oedema, cellular infiltration, goblet cell depletion, loss of architecture, crypt abscesses and serosal involvement, of which every item was subdivided as mild, moderate and severe. Incubation of colonic mucosa from rats treated with arachidonic acid and stimulated with A23187 showed an increase of the cyclooxygenase product 12-hydroxy-heptadecatrienoic acid (HHT) and the 12-1ipoxygenase product (12-HETE) and a decrease in the formation of 6-keto-prostaglandin F_1α_(6kPGF_1α_) in comparison with normal rat mucosa. Malotilate, ZY16268 and ZY16369 all resulted in a decrease in HHT, leukotriene B_4_ (LTB_4_)-like compounds and 12-hydroxyeicosaenoic acid (12-HETE) production. None of the tested compounds significantly reduced the colonic damage by acetic acid although the formation of 12-HETE was proportional to the histologically obtained inflammation score. There were marked differences in eicosanoid formation patterns between rat and human mucosa, both normal and inflamed. In view of the hyperacute nature of the mucosal damage and the marked differences in eicosanoid production, acetic acid induced colitis in rats is probably not a suitable model of ulcerative colitis in humans.


Mediators of Inflammation, 2, 67–72 (1993)

### The effect of malotilate, a derivative of malotilate and a flavenoid on eicosanoid production in inflammatory bowel disease in rats

A. P. M. van Dijk,[1] J. H. P. Wilson[2] and F. J. Zijlstra[1,CA]

[1]Department of Pharmacology, and [2]Department of Internal Medicine II, Erasmus University Rotterdam, P.O.Box 1738, 3000 DR Rotterdam, The Netherlands

[CA] Corresponding Author

**ACETIC acid induced colitis in rats was used to investigate the effects of malotilate, a drug which has been shown to inhibit 5-lipoxygenase in human macrophages, the malotilate derivate ZY16268 and the flavenoid ZY16369 on the eicosanoid production and the colonic morphology in inflammatory bowel disease. Acetic acid produced an acute inflammatory response in the colon, associated with a markedly raised inflammation score (15.8 *vs.* < 0.5), based on a seven-scaled scoring system which includes observation of haemorrhage, submucosal oedema, cellular infiltration, goblet cell depletion, loss of architecture, crypt abscesses and serosal involvement, of which every item was subdivided as mild, moderate and severe. Incubation of colonic mucosa from rats treated with arachidonic acid and stimulated with A23187 showed an increase of the cyclooxygenase product 12-hydroxy-heptadecatrienoic acid (HHT) and the 12-lipoxygenase product (12-HETE) and a decrease in the formation of 6-keto-prostaglandin $F_{1\alpha}$($6kPGF_{1\alpha}$) in comparison with normal rat mucosa. Malotilate, ZY16268 and ZY16369 all resulted in a decrease in HHT, leukotriene $B_4$ ($LTB_4$)-like compounds and 12-hydroxyeicosaenoic acid (12-HETE) production. None of the tested compounds significantly reduced the colonic damage by acetic acid although the formation of 12-HETE was proportional to the histologically obtained inflammation score. There were marked differences in eicosanoid formation patterns between rat and human mucosa, both normal and inflamed. In view of the hyperacute nature of the mucosal damage and the marked differences in eicosanoid production, acetic acid induced colitis in rats is probably not a suitable model of ulcerative colitis in humans.**

**Key words:** Eicosanoids, Experimental colitis, HPLC, Inflammation score, Rats

### Introduction

Acetic acid (HAc) colitis in rats is an experimental model of acute colitis which shares many of the histological features of ulcerative colitis such as mucosal oedema, ulceration and infiltration of the mucosa by neutrophils.[1] In contrast to ulcerative colitis which histologically is a mixture of both acute and chronic inflammation, acetic acid colitis is a pure acute inflammation. The pattern of arachidonate metabolism in HAc colitis has been reported to be similar to that in human ulcerative colitis.[1]

5-Lipoxygenase products have been proposed as major inflammatory mediators in bowel disease and in particular in ulcerative colitis.[1,2] In inflammatory bowel disease (IBD) the synthesis of leukotriene $B_4$ ($LTB_4$) is indeed increased in inflamed mucosa.[3–6] Drugs which inhibit leukotriene synthesis, such as 5-aminosalicylic acid and prednisolone, have been shown to reduce inflammation in several animal models of IBD, including acute and chronic models in rats,[7] rabbits[8] and mice.[9] In most of these studies measurements of eicosanoids were limited to $LTB_4$ and prostaglandin $E_2$ ($PGE_2$).

Apart from leukotrienes, other lipoxygenase and cyclooxygenase products are formed in inflamed tissue. Studies in which the metabolism of exogenous AA by colonic mucosa in IBD was investigated showed a three- to five-fold increase of other eicosanoids, including 12- and 15-HETE and HHT.[10] Mono-HETEs have been shown to act as stimulators of mucus secretion[11] and have inflammatory effects in the skin.[12]

Malotilate is a compound which has been reported to be effective in reducing liver damage and fibrosis in experimental liver disease and in patients with chronic hepatitis and cirrhosis.[13] It has previously been shown that malotilate inhibits lipoxygenase activity in human macrophages.[14] The suppression of 5-lipoxygenase by malotilate suggested that this or similar compounds might be

© 1993 Rapid Communications of Oxford Ltd effective anti-inflammatory agents in IBD. The authors therefore decided to study the effects of malotilate, the compound ZY 16268 (a derivate of malotilate) and ZY 16369 (a flavenoid), given systemically, in rats with HAc induced colitis. Eicosanoid synthesis in rat colonic tissue of controls, of non-treated rats with HAc colitis and of rats with HAc colitis treated with single compounds were measured. Samples were taken throughout the colon, in ascending, high sigmoid and rectum, in order to determine the predominant eicosanoids in HAc colitis, to investigate the effects of the three compounds on the eicosanoid production and to determine whether treatment with these compounds leads to a reduction in inflammation. In addition, it was decided to compare eicosanoid formation in HAc-induced colitis in rats with that found in human ulcerative proctocolitis, by examining eicosanoid formation in biopsies taken from patients undergoing diagnostic sigmoidoscopy or colonoscopy.

### Materials and Methods

*Materials:* Malotilate, ZY 16268 and ZY 16369 were provided by ZYMA, Switzerland. In view of the known difficulty in dissolving the compounds, it was decided to use polyethyleneglycol (PEG) 400 as the vehicle for administering the drugs intraperitoneally. Drugs were given at a high dose level (100 mg/kg/day) in an attempt to ensure adequate tissue levels.

*Experimental colitis:* 25 Wistar rats (Hope Farms) weighing 200 g were fasted for 36 h and then anaesthetized with ether. A midline abdominal incision was made and the junction of the coecum and ascending colon identified and ligated. Two millilitres of 5% HAc was injected into the lumen of the ascending colon through a 25 gauge needle, and this was followed immediately by an injection of 4 ml of air to clear the colon of most of the acetic acid. The incision was closed. Twenty-four hours later the rats were killed and the colons isolated. Segments approximately 5 mm long were taken for histology and for analysis of eicosanoid production from three sites—one just distal to the site of injection (ascendens), one close to the rectum and one midway in the colon (descendens).

Rats were divided into five groups:

| Group | Pre-treatment | Colon lavage |
|---|---|---|
| 1 | PEG 400 | 0.9% (w/v) NaCl |
| 2 | PEG 400 | 5.0% (w/v) HAc |
| 3 | Malotilate | 5.0% (w/v) HAc |
| 4 | ZY 16268 | 5.0% (w/v) HAc |
| 5 | ZY 16369 | 5.0% (w/v) HAc |

Malotilate, ZY 16268 and ZY 16369 were dissolved in PEG 400 and given in a dose of 100 mg/kg/day intraperitoneally for 4 days (the third administration 20 h after lavage of the colon with HAc and the last administration 1 h before the rats were killed). PEG 400 only was administered in a similar fashion to groups 1 and 2.

*Patients:* Patients fulfilling the following entry criteria were candidates for the study: (a) proven ulcerative colitis localized in the sigmoid and/or the rectum; and (b) who had no drug treatment for at least a month prior to the colonoscopy. The group consisted of eleven patients (eight men, three women, aged 21–64 years). As controls 13 patients (six men, seven women, aged 22–72 years) were taken, from whom any kind of IBD was excluded. Three biopsies were used for measurements of eicosanoid synthesis.

*Incubation conditions:* Segments of colon were weighed, minced and homogenized in 2.5 ml Krebs–Henseleit buffer pH 7.4 with an Ultra-Turrax homogenizer for 10 s on melting ice. Total protein content was determined by the Lowry method. Each sample was incubated with 0.125 $\mu$Ci [1-$^{14}$C]-archidonic acid together with 1 $\mu$M calcium ionophore A23187 (Sigma, USA) at 37°C for 15 min while shaking. After this, $^{3}$H-labelled compounds (6kPGF$_{1\alpha}$, PGF$_{2\alpha}$, PGE$_2$, TxB$_2$, LTB$_4$, 15-HETE, 12-HETE and 5-HETE) were added as chromatographic standards and for recovery purposes (all labelled compounds were from Amersham, UK). Samples were centrifuged for 2 min at 16 000 × *g* at 4°C. The supernatant was applied to a Sep Pak C$_{18}$ cartridge (Waters Ass., USA), eluted with methanol and dried with a Savant Speed Vac concentrator. The pellet was dissolved in 250 $\mu$l methanol and filtered through an Anatop 0.2 $\mu$m filter into an HPLC polypropylene microvial. One hundred microlitres of this was injected onto two combined Nucleosil 5C$_{18}$ HPLC columns (3 × 200 mm, Chrompack, The Netherlands). HPLC was performed with a Hewlett Packard 1048B liquid chromatograph with variable wavelength detector. Radioactivity was measured on-line with a Berthold LB506C monitor. The solvent system contained a gradient of 0.12% trifluoracetic acid and 0.2% triethylamine in water (pH 3.0) and acetonitrile (LichrsolvR, Merck, Germany). The flow rate was 0.5 ml/min at 37°C.[15]

*Histological studies:* Representative cross-sections, taken from the rat colon at 1, 6 and 12 cm distal to the coecal-ascending colon junction, were fixed in 4% buffered formaldehyde. Histological examinations were performed with haematoxylin and azafloxin stained cross-sections of the colons. The severity of colitis was graded by a seven-scaled scoring system which includes observation of haemorrhage, submucosal oedema, cellular infiltration, goblet cell depletion, loss of architecture, crypt abscesses and serosal involvement, of which every item was scored as mild, moderate and severe. Therefore, there was a minimum histological grade of 0 and a maximum grade of 21.

*Statistical analysis:* Data are reported as the mean ± S.E.M. of the combined experiments. Differences were analyzed for significance using Student's two tailed *t* test, the Wilcoxon's test for paired variates and the Duncan's multiple range test. Values of $p < 0.05$ were considered significant.

### Results

*Weight:* The mean wet weight of the sections of all the groups were: in the ascendens, 161 ± 9 mg; in the descendens, 131 ± 8 mg; and in the rectum, 109 ± 5 mg. Protein content was increased in the descendens and the rectum in comparison with the ascending colon (ascendens 60 ± 3 μg/mg, descendens 67 ± 3 μg/mg, rectum 73 ± 3 μg/mg).

*Eicosanoid formation:* The pattern of the most common eicosanoids formed by rat rectal colonic tissue are shown in Fig. 1a, and the eicosanoid formation by human rectal mucosa is given in Fig. 1b. The pattern of eicosanoids produced by rat colonic tissue, after incubation with $^{14}$C-AA and stimulation by A23187 differs from that produced by humans. In rats the main metabolites of AA are 6kPGF$_{1\alpha}$ (48.4%), 12-HETE (15.5%) and HHT (17.5%), whereas the main products of human rectal mucosa are 15-HETE (32.0%), HHT (18.6%), TxB$_2$ (12.0%) and PGE$_2$ (10.8%).

Induction of an acute colitis in rats with HAc results in a significant increase in 12-HETE (32.9%) formation and a significant decrease in the formation of 6kPGF$_{1\alpha}$ (22.1%). In ulcerative colitis in men only 15-HETE was significantly increased. In both humans and rats, LTB$_4$-like compounds represent a minor part of the AA metabolism.

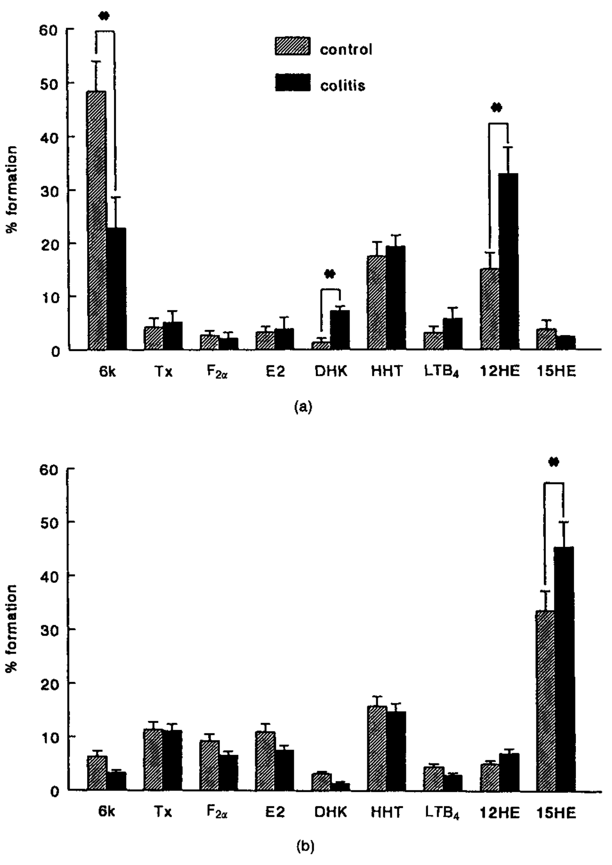

FIG. 1. (a) Formation of the most common eicosanoids of rat rectal mucosa, after either saline (mean total formation 8100 dpm/100 μg protein) or acetic acid treatment (mean total formation 24870 dpm/100 μg protein); and (b) of non-inflamed (mean total formation 6890 dpm/100 μg protein) and inflamed (mean total 7990 dpm/100 μg protein) human mucosa, expressed as percentage of total formation (*$p < 0.05$ *vs.* controls).

Significant changes in the individual eicosanoids following drug treatment were only seen in the rectum. In Table 1 the formation of some eicosanoids by rectal tissue is given for malotilate and the compounds ZY 16268 and ZY 16369 in comparison with the non-treated HAc induced colitis. Table 1 shows that malotilate, ZY 16268 and ZY 16369 all resulted in a decrease in the HHT,

**Table 1** Formation of eicosanoids by rat rectal colonic tissue *vs.* treatment with or without different compounds (expressed as dpm/100 μg protein)

| Group ($n = 5$) | Eicosanoids formed | | | |
|---|---|---|---|---|
| | 6kPGF$_{1\alpha}$ | LTB$_4$ | HHT | 12-HETE |
| Control | 3240 ± 980 | 910 ± 240 | 1160 ± 100 | 2350 ± 470 |
| Non-treated HAc colitis | 5720 ± 2220 | 1600 ± 340* | 4170 ± 380* | 7500 ± 1760* |
| Malotilate HAc colitis | 5460 ± 190 | 720 ± 330** | 1540 ± 140** | 3250 ± 370** |
| ZY 16268 HAc colitis | 5610 ± 1120 | 680 ± 100** | 2760 ± 540** | 4060 ± 470** |
| ZY 16369 HAc colitis | 6390 ± 940 | 580 ± 70** | 2810 ± 210** | 5440 ± 590 |

* $p < 0.05$ *vs.* controls.
** $p < 0.05$ *vs.* non-treated HAc colitis.

**Table 2** Mean inflammation score after treatment with HAc, malotilate, ZY 16268 and ZY 16369

| Group ($n = 5$) | Score |
|---|---|
| Control | <0.5 |
| Non-treated HAc colitis | $15.8 \pm 1.9$ |
| Malotilate HAc colitis | $14.7 \pm 2.8$ |
| ZY 16268 HAc colitis | $17.2 \pm 1.2$ |
| ZY 16369 HAc colitis | $13.8 \pm 2.1$ |

$LTB_4$ and 12-HETE production. The three compounds inhibit both the 5- and 12-lipoxygenase products and HHT.

*Inflammation score:* HAc produced a marked inflammatory response in the mucosa and the submucosa colon, associated with a significantly raised inflammation score (mean 15.8 *vs.* < 0.5). Although the formation of the 5- and 12-lipoxygenase products was significantly inhibited, none of the tested compounds had significant effect on the inflammation score. The compound ZY 16268 resulted in a slight increase in inflammation score (17.2 *vs.* 15.8) (Table 2). The 12-HETE production showed a significant correlation (rs: 0.58; $p < 0.01$) with the inflammation scores (Fig. 2).

### Discussion

The present study was undertaken to investigate the effect of malotilate, its derivate ZY 16268 and flavenoid ZY 16369 on the eicosanoid production in a rat model of acute colonic inflammation. It was decided to use the model acetic acid induced colitis in rats, because of the expected similarity of the

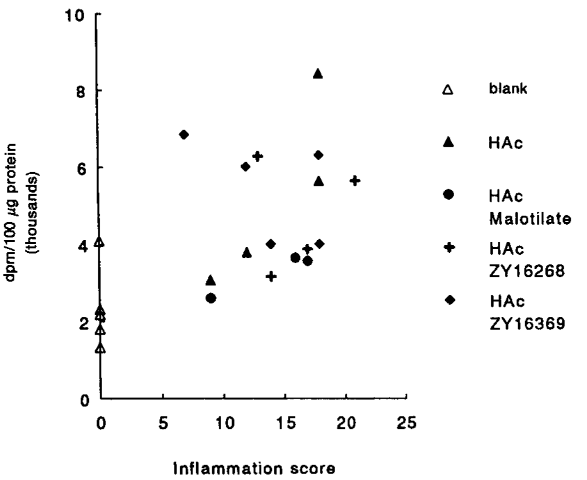

FIG. 2. Inflammation score *vs.* 12-HETE production (dpm/100 µg protein) by rat rectal colonic tissue ($n = 22$, $p < 0.01$, $r = 0.58$).

pattern of arachidonate metabolism to human IBD.[1] This rat model provoked a severe inflammation of the colon, in which a mean of 80% of the maximal inflammation score was reached. In the normal colonic mucosa of rats, relatively high amounts of $6kPGF_{1\alpha}$, HHT and 12-HETE were formed. Previous experiments have shown that 5-HETE is also one of the prominent products.[1]

After induction of the inflammation, total eicosanoid production was dramatically increased, although the relative formation did not change significantly. In HAc colitis mucosa a significant increase of $LTB_4$, HHT and 12-HETE compared to normal rat mucosa was observed. In inflamed colonic mucosa of patients with active ulcerative colitis, the synthesis of leukotrienes, products of 5-lipoxygenase metabolism and prostaglandins (PGs), products of the cyclooxygenase metabolism are enhanced, compared to normal tissue.[1–3,16] Recently, the authors have shown that the 15-lipoxygenase product 15-HETE is the main eicosanoid formed by human colonic mucosa and that this metabolite of AA is increased significantly in inflamed human colonic mucosa.[17]

The results of this study indicate that the pattern of eicosanoid formation in HAc induced colitis in rats differs from those seen in human IBD. This is in agreement with our previous findings in which marked species differences in the pattern of eicosanoid formation by macrophages were observed.[18]

The only AA metabolite positively correlated with the inflammation score was 12-HETE. The same was observed with 15-HETE in a human IBD study.[17] The functions of 12- and 15-HETE in inflamed colonic mucosa is not clear. In blood platelets, the major AA metabolites are $TxB_2$, HHT and 12-HETE,[19] the same eicosanoids that are increased in acetic acid colitis mucosa. Macrophages mainly form $LTB_4$ and 5-HETE,[14] whereas endothelial cells generate $PGI_2$ (prostacyclin) and 15-HETE. It is possible that whether endothelial cells in the colonic mucosa synthesize either $PGI_2$ (measured as 6-keto-$PGF_{1\alpha}$) or 15-HETE is species dependent. Furthermore the origin of these cells (alveoli, liver, kidney or colon) could restrict the differential synthesis of eicosanoids.

Histological examination of the rectal and sigmoidal colonic tissues showed a number of haemorrhages, which suggests the participation of platelets and endothelial cells in both the inflammation and the eicosanoid formation (Fig. 3). The present investigations showed that treatment with malotilate, ZY 16268 or ZY 16369 resulted in a significant decrease of the production of HHT and $LTB_4$. Both malotilate and its derivate ZY 16268 gave a significant reduction in the formation of 12-HETE. The effects of the three

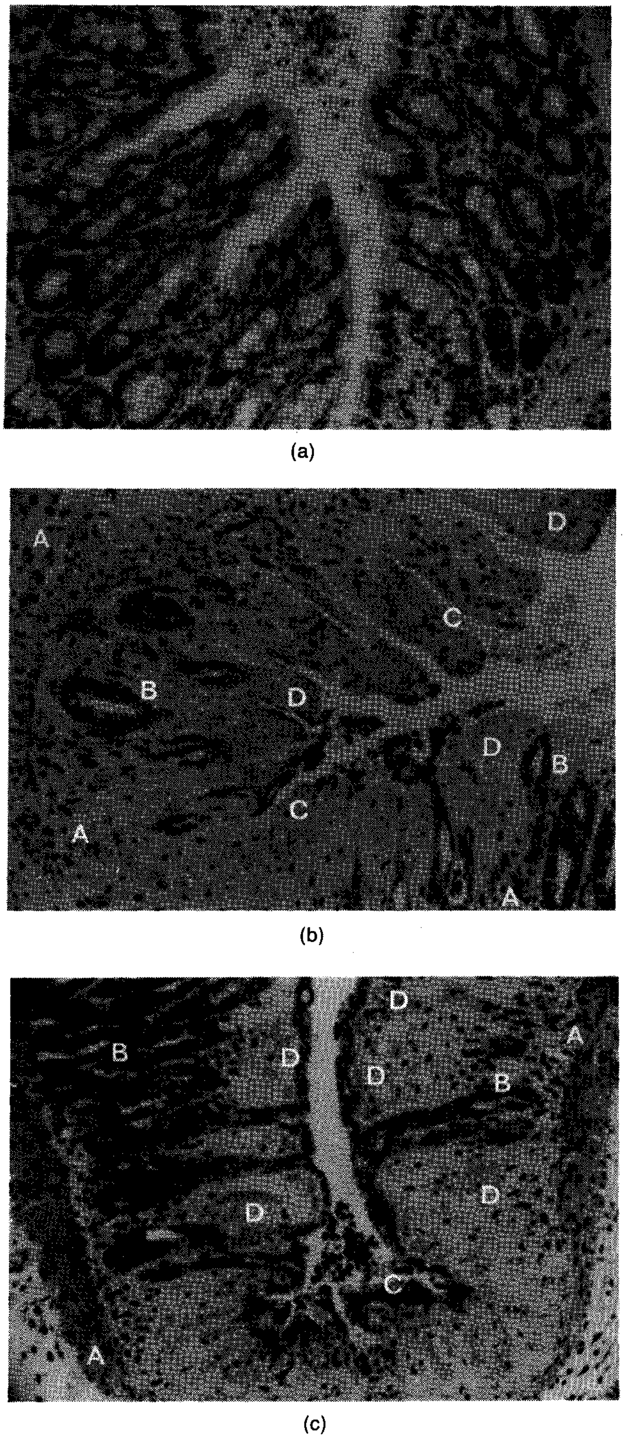

FIG. 3. Histological sections of rectal colonic mucosa. Tissue samples were taken and fixed immediately after sacrifice. (a) Section from a control rat—no histological detectable damage was observed; (b) sections of rectum after non-treated acetic acid; and (c) ZY 16369 treated acetic acid showing cell infiltration (A), goblet cell depletion (B), loss of architecture (C) and areas of haemorrhages (D). (×250).

drugs on eicosanoid production are similar to those previously reported with human peritoneal macrophages, with the exception of malotilate which showed an increase of the 12-HETE production, human macrophages, possibly due to a positive feedback mechanism.[20]

In ulcerative colitis the initial event is followed by a secondary reaction, in which inflammatory cells secrete mediators, with both beneficial and harmful effects. From our investigations it is clear that, although the formation of mediators of inflammation was successfully inhibited, the degree of inflammation did not improve. This unexpected and contradictory finding could be due to: (i) Mediators of inflammation such as eicosanoids do not play an important role in the *initial* phase of the inflammation. Recent evidence suggests a key role of cytokines and platelet activating factor (PAF)[21] in the initial stages, whereas eicosanoids could be important regulators during the later (chronic) inflammation. (ii) This type of experimental colitis is too acute (<24 h) for a major role for inflammatory cells, which are characteristic of later stages of inflammation. The evidence for this is the low number of leucocytes and macrophages, the main donors for eicosanoid production. A subacute model for colitis, such as the dextran sodium sulphate (DSS) mouse model[9] or the TNBS (2,4,4-trinitrobenzene sulphonic acid) rat model[22] would probably be a better choice to observe the effects of potent 5-lipoxygenase synthesis inhibitors on *in vivo* inflammation.

### References

1. Sharon P, Stenson WF. Metabolism of arachidonic acid in acetic acid colitis in rats. *Gastroenterology* 1985; **88:** 55–63.
2. Donowitz M. Arachidonic acid metabolites and their role in inflammatory bowel disease. *Gastroenterology* 1985; **88:** 580–587.
3. Schumert R, Towner J, Zipser RD. Role of eicosanoids in human and experimental colitis. *Dig Dis Sci* 1988; **33:** 58S–64S.
4. Lauritsen K, Laursen LS, Bukhave K, Rask-Madsen J. Intra-luminal colonic levels of arachidonic acid metabolites in ulcerative colitis. *Adv Prost Thromb Leukotr Res* 1987; **17:** 347–352.
5. Lauritsen K, Laursen LS, Bukhave K, Rask-Madsen J. Use of colonic eicosanoid concentrations as predictors of relapse in ulcerative colitis: double blind placebo controlled study on sulphasalazine maintenance treatment. *Gut* 1988; **29:** 1316–1321.
6. Lauritsen K, Laursen LS, Bukhave K, Rask-Madsen J. Effects of topical 5-aminosalicylic acid and prednisolone on prostaglandin $E_2$ and leukotriene $B_4$ levels determined by equilibrium *in vivo* dialysis of rectum in relapsing ulcerative colitis. *Gastroenterology* 1986; **91:** 837–844.
7. Wallace JL, MacNaughton WK, Morris GP, Beck PL. Inhibition of leukotriene synthesis markedly accelerates healing in a rat model of inflammatory bowel disease. *Gastroenterology* 1989; **96:** 29–36.
8. Zipser RD, Nast CC, Lee M, Kao HW, Duke R. *In vivo* production of leukotriene $B_4$ in rabbit colitis. *Gastroenterology* 1987; **92:** 33–39.
9. Okayasu I, Hatakeyama S, Yamada M, Ohkusa T, Inagaki Y, Nakaya R. A novel method in the induction of reliable experimental acute and chronic ulcerative colitis in mice. *Gastroenterology* 1990; **98:** 694–702.
10. Sharon P, Stenson WF. Enhanced synthesis of leukotriene $B_4$ by colonic mucosa in inflammatory bowel disease. *Gastroenterology* 1984; **85:** 453–460.
11. Johnson HG, McNee ML, Sun FF. 15-Hydroxyeicosatetraenoic acid is a potent inflammatory mediator and agonist of canine tracheal mucus secretion. *Am Rev Respir Dis* 1985; **131:** 917–922.
12. Higgs GA, Salmon JA, Spayne JA. The inflammatory effects of hydroperoxy and hydroxy acid products of arachidonate lipoxygenase in rabbit skin. *Br J Pharmacol* 1981; **74:** 429–433.
13. Ryle PR, Dumont JM. Malotilate: The new hope for a clinically effective agent for the treatment of liver disease. *Alcohol Alcoholism* 1987; **22:** 121–141.
14. Zijlstra FJ, Wilson JHP, Vermeer MA, Ouwendijk RJTh, Vincent JE. Differential effects of malotilate on 5-, 12-, and 15-lipoxygenase in human ascites cells. *Eur J Pharmacol* 1989; **159:** 291–295.
15. Ham Van der AC, Kort WJ, Byma AM, Zijlstra FJ, Vermeer MA, Jeekel J. Eicosanoid profile of healing colon anastomosis and peritoneal macrophages in the rat. *Gut* 1990; **31:** 807–811.
16. Schumert R, Towner J, Zipser RD. Role of eicosanoids in human and experimental colitis. *Dig Dis Sci* 1988; **33:** 58S–64S.

17. Zijlstra FJ, Dijk van APM, Wilson JHP, Riemsdijk-van Overbeek IC, Vincent JE, Ouwendijk RJTh. 15-HETE is the main eicosanoid formed by human colonic mucosa. *Agents Actions* 1992; **35:** C53–C59.
18. Ouwendijk RJTh, Zijlstra FJ, Broek van den AMWC, Brouwer A, Wilson JHP, Vincent JE. Comparison of the production of eicosanoids by human and rat peritoneal macrophages and rat Kuppfer cells. *Prostaglandins* 1988; **35:** 437–446.
19. Vincent JE, Zijlstra FJ, Vliet van H. Determination of the formation of $TxB_2$, HHT and 12-HETE from arachidonic acid and of the $TxB_2$:HHT, $TxB_2$:HETE and ($TxB_2$ + HHT): HETE ratio in human platelets. Possible use in diagnostic purposes. *Prostaglandins Med* 1980; **5:** 79–84.
20. Montandon JB, Zijlstra FJ, Wilson JHP, Grandjean EM, Cicurel L. *In vitro* versus *in vivo* activities of new 5-lipoxygenase inhibitors with anti-inflammatory activity. *Int J Tissue React* 1989; **11:** 107–112.
21. Wallace JL. Release of platelet-activating factor (PAF) and accelerated healing induced by a PAF antagonist in an animal model of chronic colitis. *Can J Physiol Pharmacol* 1988; **66/4:** 422–425.
22. Selve N, Wöhtmann T. Intestinal inflammation in TNBS sensitised rats as a model of chronic inflammatory bowel disease. *Mediators of Inflammation* 1992: **1:** 121–126.

**Received 27 November 1992; accepted in revised form 8 December 1992**
